# Outcomes in patients with clinically suspected pedal osteomyelitis based on bone marrow signal pattern on MRI

**DOI:** 10.5194/jbji-8-99-2023

**Published:** 2023-03-22

**Authors:** Christin A. Tiegs-Heiden, Tanner C. Anderson, Mark S. Collins, Matthew P. Johnson, Douglas R. Osmon, Doris E. Wenger

**Affiliations:** 1 Department of Radiology, Mayo Clinic, Rochester, MN, USA; 2 Department of Biomedical Statistics and Informatics, Mayo Clinic, Rochester, MN, USA; 3 Department of Public Health, Infectious Diseases, and Occupational Medicine, Mayo Clinic, Rochester, MN, USA

## Abstract

**Objective**: confluent T1 hypointense marrow signal is widely accepted to represent osteomyelitis on MRI. Some authors have suggested that non-confluent bone marrow signal abnormality should be considered early osteomyelitis. The purpose of this study was to address this issue by comparing the rate of osteomyelitis and amputation based on T1 marrow signal characteristics. **Materials and methods**: a total of 112 patients who underwent MRI of the foot for the evaluation of possible osteomyelitis were included. Patients were assigned to confluent T1 hypointense, reticulated T1 hypointense, and normal bone marrow signal groups. **Results**: patients with confluent T1 hypointense signal on MRI had significantly higher rates of osteomyelitis and amputation at 2 and 14 months post-MRI than the reticulated T1 hypointense group ( p<0.001 ). Six patients had normal T1 signal, 16.7 % of whom had osteomyelitis and underwent amputation by 2 months post-MRI. Of 61 patients with reticulated T1 hypointense signal, 19.7 % had a diagnosis of osteomyelitis at 2 months post-MRI and 30.8 % had a diagnosis of osteomyelitis at 14 months post-MRI; moreover, 14.8 % and 31.5 % underwent amputation by 2 and 14 months post-MRI, respectively. Of 45 patients with confluent T1 hypointense signal, 73.3 % of patients had osteomyelitis at 2 months post-MRI and 82.5 % had osteomyelitis at 14 months post-MRI. In this group, 66.7 % underwent amputation by 2 months post-MRI and 77.8 % underwent amputation by 14 months post-MRI. **Conclusions**: over half of the patients with suspected pedal osteomyelitis who had reticulated or normal T1 bone marrow signal on MRI healed with conservative measures. Therefore, we recommend terminology such as “osteitis”, “reactive osteitis”, or “nonspecific reactive change” to describe bone marrow edema-like signal and reticulated hazy T1 hypointense signal without associated confluent T1 hypointensity. Moreover, we recommend that the MRI diagnosis of osteomyelitis is reserved for confluent T1 hypointense bone signal in the area of concern.

## 1 Introduction

Pedal soft tissue ulcers are a common complication in patients with diabetes and/or peripheral vascular disease. Up to 80 % of these ulcers will become infected, and this infection leads to osteomyelitis about 20 % of the time (Geraghty and Laporta, 2019). Patients with osteomyelitis may have worse outcomes, including longer hospital stays, longer duration of or different types of antimicrobial therapy (IV vs. oral), longer time to wound healing, and a greater rate of amputation (Mutluoglu et al., 2013). The spectrum of treatment options for pedal osteomyelitis ranges from conservative treatment with systemic or local antimicrobial therapy to amputation (Senneville and Robineau, 2017). In the setting of infection or critical limb ischemia, the amputation rate is 50 %–60 % (Lipsky et al., 2013; Lipsky, 2004). Patients with a diabetic foot ulcer have a 3-year cumulative mortality rate of 28 %, which increased to over 80 % following a major (above ankle) amputation in one study (Geraghty and Laporta, 2019; Vuorlaakso et al., 2021).

Imaging plays an important role in the diagnosis of osteomyelitis. Although plain radiographs should be obtained as the initial imaging study, MRI is the most sensitive modality for the identification of osteomyelitis (Lee et al., 2016). As such, MRI is often useful in cases of suspected osteomyelitis with negative or inconclusive radiographs. The MRI appearance of increased intramedullary signal on fluid-sensitive sequences with associated confluent T1 hypointensity is widely accepted to be consistent with a diagnosis of osteomyelitis (Toledano et al., 2011; Alaia et al., 2021; Johnson et al., 2009). A recent study of MRI findings of osteomyelitis in the long bones found that only 4 % of patients with osteomyelitis lacked confluent T1 hypointense marrow signal on MRI (Crim et al., 2022). The finding of increased intramedullary signal in the area of concern on fluid-sensitive sequences with hazy reticulated (non-confluent) T1 hypointense signal, however, has recently come into question and can present a diagnostic challenge. One study of patients with pedal ulcers and underlying T2 hyperintense marrow signal found that 61 % of these patients progressed to osteomyelitis by 14 months (Duryea et al., 2017). That study therefore suggested that abnormal hazy T1 hypointense and T2 hyperintense bone marrow signal be considered early osteomyelitis (Duryea et al., 2017). Another study found that patients with osteomyelitis almost always had T1 hypointense bone marrow signal, whereas reticulated T1 signal was seen nearly evenly between patients with and without osteomyelitis (Jang et al., 2020). A recent review article proposed the term “infectious osteitis” for these cases with non-confluent T1 signal abnormalities (Alaia et al., 2021).

The distinction between these terms is important. Although only one of many factors, an MRI diagnosis of osteomyelitis or “early osteomyelitis” could potentially increase the likelihood of more aggressive treatment, such as amputation. No mention of osteomyelitis or infectious osteitis in the MRI report may argue in favor of an attempt at conservative treatment such as local debridement combined with antimicrobial therapy. In order to select the most appropriate terminology for various MRI findings, it is essential that the relationship between MRI appearance and clinical outcomes is understood.

The purpose of the current study was to assess the relationship between T1 bone marrow signal characteristics on pedal MRI and clinical outcomes, specifically the rate of progression to osteomyelitis and amputation. We hypothesize that patients with confluent T1 hypointense marrow replacing signal abnormality are more likely to fail conservative management and require earlier amputation than those with normal or hazy T1 signal.

## 2 Materials and methods

Institutional review board (IRB) approval was obtained prior to performing this retrospective research study, and the requirement for informed patient consent was waived.

**Figure 1:**
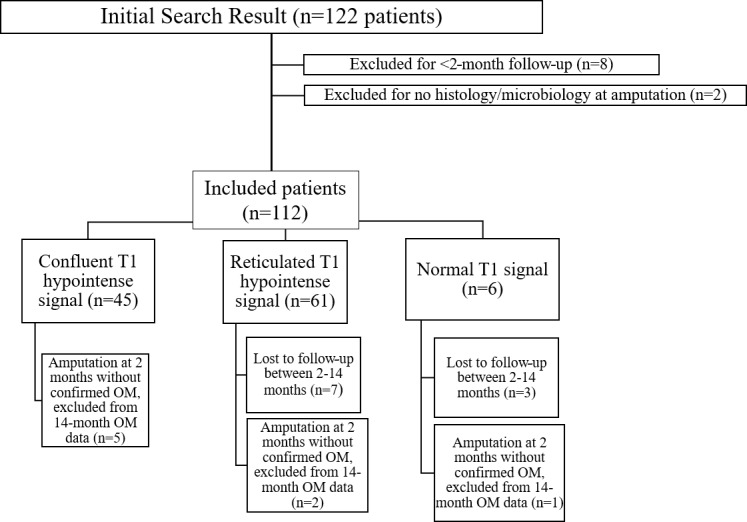
Patient selection flowchart (OM denotes osteomyelitis).

A search engine at our institution was used to identify foot MRI examinations performed between 20 February 2015 and 6 January 2020 for the evaluation of possible osteomyelitis. Patients over age 17 who underwent MRI of the foot for a clinical question of osteomyelitis were included. Patients with less than 2 months of clinical follow-up were excluded (eight patients). Patients who underwent amputation after the MRI but did not have histology or microbiology available from the procedure were excluded. A patient selection flowchart is shown in Fig. 1.

For all patients, the medical record was reviewed for age, sex, diabetic status, presence of peripheral vascular disease, and the presence or absence of a skin ulcer. Any available tissue histology, microbiology, or blood cultures were recorded, and relevant clinical and imaging follow-up information was included. Treatment, such as oral or intravenous (IV) antibiotics or amputation within 1 week of the initial MRI, was recorded. Amputation of the affected body part and the presence or absence of osteomyelitis were documented at 2 months post-MRI and again 1 year later at 14 months post-MRI, when available. A diagnosis of osteomyelitis (as defined below and as per prior studies such as Duryea et al., 2017, and Jang et al., 2020) or amputation that occurred at any point in the time before the respective follow-up time point was included. Any patient that had an amputation at 2 months post-MRI without a confirmed diagnosis of osteomyelitis per the criteria above at that time was subsequently removed from the 14-month osteomyelitis analysis.

The diagnosis of osteomyelitis was made as follows. Patients who underwent amputation or surgical debridement with histologic examination consistent with osteomyelitis were diagnosed as such. When a surgical tissue diagnosis was not available, patients were considered positive for osteomyelitis if they had a positive blood culture and radiographic progression, imaging progression on a subsequent MRI, or lack of improvement with conservative clinical management (soft tissue debridement or antibiotic therapy). Patients were considered negative for osteomyelitis if they responded to conservative management (soft tissue debridement or antimicrobial therapy) and did not subsequently relapse.

All of the MRI examinations were performed on 1.5 or 3 Tesla GE Healthcare (Chicago, Illinois, USA) MRI scanners at our institution (Mayo Clinic). A dedicated foot and ankle extremity or knee coil was used in all cases. Images were obtained in three orthogonal planes of T1 and short-TI inversion recovery (STIR) or T2 fat-saturated sequences. T1-weighted fast spin-echo images were performed with the following parameters: repetition time/time to echo (TR/TE) of 700–900/10–20, 4 mm thick in the axial plane and 3 mm thick in coronal and sagittal planes, 0 mm skip, 384×280 matrix, 1 number of excitations (NEX), and a 2–4 echo train length (ETL). STIR images were performed with the following parameters: TR/TE of 3000–6000/45, 4 mm thick in the axial plane and 3 mm thick in coronal and sagittal planes, 0 mm skip, 384×280 matrix, 1 NEX, and an 8–10 ETL. T2 fat-saturated conventional spin-echo images were performed with the following parameters: TR/TE of 3000–6000/45, 4 mm thick in the axial plane and 3 mm thick in coronal and sagittal planes, 0 mm skip, 384×224 matrix, 3 NEX, and an 8–10 ETL. Contrast-enhanced imaging is not typically performed at our institution for the evaluation of osteomyelitis and was not included in the current study.

**Figure 2:**
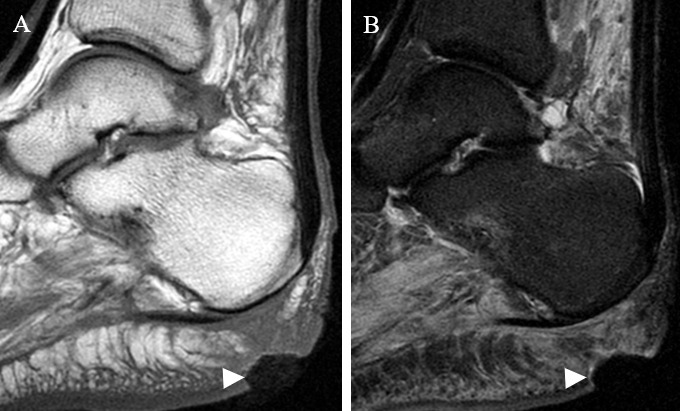
Sagittal T1 **(a)** and STIR **(b)** sequences in a 46-year-old female patient who underwent MRI for the evaluation of osteomyelitis. There is a prominent cutaneous ulcer (arrowhead) with underlying soft tissue changes that extend to bone. The calcaneal bone marrow signal is normal. This patient improved with conservative measures. A follow-up MRI (not shown) demonstrated resolution of the ulcer with normal calcaneal bone marrow.

**Figure 3:**
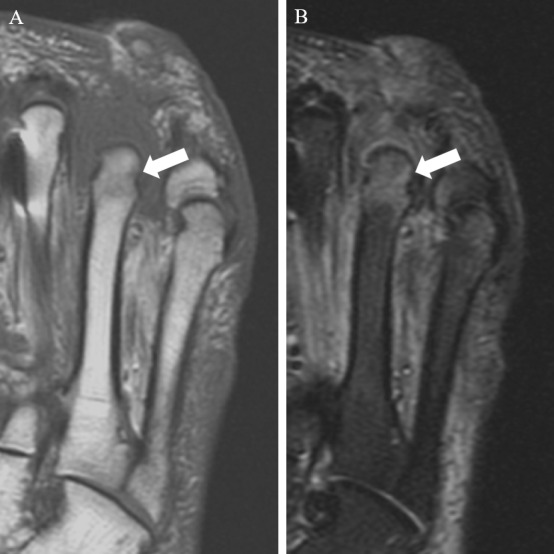
Coronal T1 **(a)** and STIR **(b)** images of the forefoot in a 45-year-old male with prior fourth- and fifth-toe amputations and recurrent ulcer. There is hazy reticulated T1 hypointense signal within the fourth metatarsal head, with relative preservation of marrow fat signal (arrow in panel **a**). STIR images demonstrate hyperintensity in this area (arrow in panel **b**).

**Figure 4:**
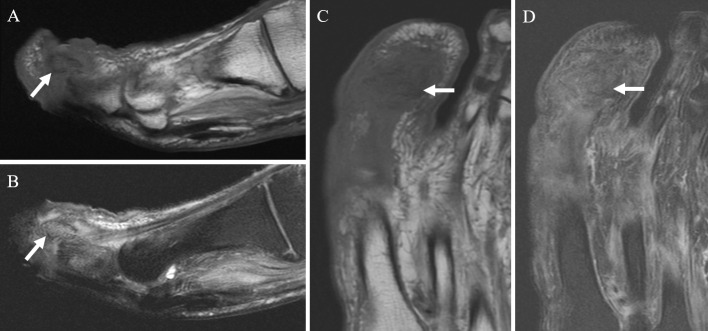
Sagittal T1 **(a)** and T2 fat-saturated **(b)** and coronal T1 **(c)** and T2 fat-saturated **(d)** images in a 55-year-old male patient with a great toe ulcer demonstrate confluent T1 hypointense and T2 hyperintense signal in the first distal phalanx (arrows), consistent with osteomyelitis.

All MRI examinations were blindly reviewed by a subspecialty trained musculoskeletal radiologist with 6 years' experience. MR images were viewed on a high-resolution picture archiving and communication system station (Visage Imaging, Richmond Australia). The osseous marrow signal was reviewed in the area of concern. Marrow signal on T1-weighted images was classified into one of three patterns: normal T1 signal (Fig. 2), hazy reticulated T1 hypointense signal (Fig. 3), or confluent geographic T1 hypointense signal (Fig. 4). Reticulated T1 hypointense signal was defined as a hazy decrease in marrow fat signal with some interspersed normal marrow fat. Confluent T1 hypointense signal was defined as decreased signal intensity with complete replacement of the bone marrow fat. The presence of a skin ulcer was also noted (yes/no). Subsequently, the classification of marrow signal from this retrospective review was compared to the initial radiology report by the author who performed chart review. If the blinded review and original report matched, it was classified as such; if not, the final determination was made by a subspecialty trained musculoskeletal radiologist with 24 years' experience.

### Statistical methods

Distributions of quantitative variables were assessed for unimodality, symmetry, and outliers prior to analysis. Continuous variables were summarized as the mean with the standard deviation and range and were compared between groups using a one-way ANOVA. Categorical variables were summarized as the total with a percentage and were compared between groups using a chi-squared goodness-of-fit test or Fisher's exact test. Post hoc pairwise comparison testing using the chi-squared test was performed between the confluent and reticulated and between the reticulated and normal groups. This post hoc comparison was considered descriptive and was not adjusted for multiple testing. All p values of less than 0.05 were considered statistically significant throughout the analysis. The statistical analyses were completed using R version 4.0.3 (R Foundation for Statistical Computing, Vienna, Austria) with the tidyverse 1.3.1 package suite and the arsenal 3.6.3 package.

## 3 Results

The patient selection flowchart is shown in Fig. 1. Clinical follow-up was available for all patients at 2 months. A total of 10 patients (8.9 %) did not have a follow-up at 14 months and, therefore, were not included in the analysis at that time point: 3 patients from the normal T1 signal group and 7 patients from the reticulated signal group.

This study included 112 patients, 32 of which were female (28.6 %). The mean age of the patients was 62 years (range of 26–98 years, with a standard deviation of 16.16 years). There were 6 patients in the normal T1 bone marrow signal group, 61 patients in the reticulated T1 hypointense group, and 45 patients in the confluent T1 hypointense group. There was no significant difference in age, sex, presence of a skin ulcer, diagnosis of diabetes mellitus, diagnosis of peripheral vascular disease, or antibiotic use between the three T1 signal groups (Table 1). When ulcers were present, they were located in the following portions of the foot: forefoot – 64 of 93 patients (68.8 %); mid-foot – 22 of 93 patients (23.7 %); and hindfoot – 7 of 93 patients (7.5 %).

**Table 1:** Patient demographics, in total and for each T1 signal group.

	Total ( N=112 )	Normal T1 ( N=6 )	Reticulated T1 ( N=61 )	Confluent T1 ( N=45 )	p value
Age					0.987
Mean (SD)	61.75 (15.61)	61.50 (13.84)	61.97 (15.60)	61.49 (16.16)	
Range	26–98	46–85	27–86	26–98	
Sex					0.800
F	32 (28.6 %)	1 (16.7 %)	18 (29.5 %)	13 (28.9 %)	
M	80 (71.4 %)	5 (83.3 %)	43 (70.5 %)	32 (71.1 %)	
Diabetes					0.992
No	23 (20.5 %)	1 (16.7 %)	12 (19.7 %)	10 (22.2 %)	
Yes	89 (79.5 %)	5 (83.3 %)	49 (80.3 %)	35 (77.8 %)	
PVD					0.087
No	56 (50.0 %)	5 (83.3 %)	33 (54.1 %)	18 (40.0 %)	
Yes	56 (50.0 %)	1 (16.7 %)	28 (45.9 %)	27 (60.0 %)	
Ulcer					0.162
No	19 (17.0 %)	1 (16.7 %)	14 (23.0 %)	4 (8.9 %)	
Yes	93 (83.0 %)	5 (83.3 %))	47 (77.0 %)	41 (91.1 %)	
Antibiotic					0.393
No	19 (17.1 %)	4 (66.7 %)	14 (23.3 %)	4 (8.9 %)	
Oral	20 (18.0 %)	1 (16.7 %)	9 (15.0 %)	10 (22.2 %)	
IV	72 (64.9 %)	1 (16.7 %)	37 (61.7 %)	31 (68.9 %)	

The abbreviations used in the table are as follows: SD – standard deviation, F – female, M – male, PVD – peripheral vascular disease, and IV – intravenous.

Rates of amputation and osteomyelitis, in total and for each MRI group, are shown in Table 2. Multivariate analysis showed a significant difference in the rate of osteomyelitis and amputation between bone marrow signal groups at the 2- and 14-month time points ( p<0.001 ).

In cases of confluent geographic T1 hypointense signal abnormality, 30 of 35 (85.7 %) amputations occurred within 2 months. In cases of reticulated or normal T1 signal, 10 of 18 (55.6 %) amputations occurred within 2 months.

**Table 2:** Rate of amputation and osteomyelitis at 2 and 14 months, in total and for each T1 signal group.

	Total	Normal T1	Reticulated T1	Confluent T1	p value
	( N=112 )	( N=6 )	( N=61 )	( N=45 )	
Amputation at 2 months					<0.001
No	72 (64.3 %)	5 (83.3 %)	52 (85.2 %)	15 (33.3 %)	
Yes	40 (35.7 %)	1 (16.7 %)	9 (14.8 %)	30 (66.7 %)	
Amputation at 14 months	( N=102 )	( N=3 )	( N=54 )	( N=45 )	<0.001
No	49 (48.0 %)	2 (66.7 %)	37 (68.5 %)	10 (22.2 %)	
Yes	53 (52.0 %)	1 (3.3 %)	17 (31.5 %)	35 (77.8 %)	
Osteomyelitis at 2 months	( N=112 )	( N=6 )	( N=61 )	( N=45 )	<0.001
No	66 (58.9 %)	5 (83.3 %)	49 (80.3 %)	12 (26.7 %)	
Yes	46 (41.1 %)	1 (16.7 %)	12 (19.7 %)	33 (73.3 %)	
Osteomyelitis at 14 months	( N=96 )	( N=4 )	( N=52 )	( N=40 )	<0.001
No	46 (47.9 %)	3 (75.0 %)	36 (69.2 %)	7 (17.5 %)	
Yes	50 (52.1 %)	1 (25.0 %)	16 (30.8 %)	33 (82.5 %)	

Results of post hoc pairwise testing between the geographic confluent T1 hypointense and reticulated T1 hypointense intramedullary signal abnormality groups are shown in Table 3. There was a significant difference between these groups with respect to the rate of both confirmed osteomyelitis and amputation at the 2- and 14-month time frames.

There was no significant difference in amputation between the reticulated hypointense and normal T1 signal groups at 2 months (9 of 61 cases (14.8 %) vs. 1 of 6 cases (16.7 %); p=1.000 ). There was also no significant difference in osteomyelitis at 2 months (12 of 61 cases (19.7 %) vs. 1 of 6 cases (16.7 %); p=1.000 ). There were not enough data at the 14-month time point to assess for statistical significance between these two groups.

**Table 3:** Post hoc pairwise testing between the confluent and reticulated T1 bone marrow signal groups.

	Reticulated ( N=61 )	Confluent ( N=45 )	p value
Amputation at 2 months			<0.001
No	52 (85.2 %)	15 (33.3 %)	
Yes	9 (14.8 %)	30 (66.7 %)	
Amputation at 14 months			<0.001
No	37 (68.5 %)	10 (22.2 %)	
Yes	17 (31.5 %)	35 (77.8 %)	
Osteomyelitis at 2 months			<0.001
No	49 (80.3 %)	12 (26.7 %)	
Yes	12 (19.7 %)	33 (73.3 %)	
Osteomyelitis at 14 months			<0.001
No	36 (69.2 %)	7 (17.5 %)	
Yes	16 (30.8 %)	33 (82.5 %)	

**Figure 5:**
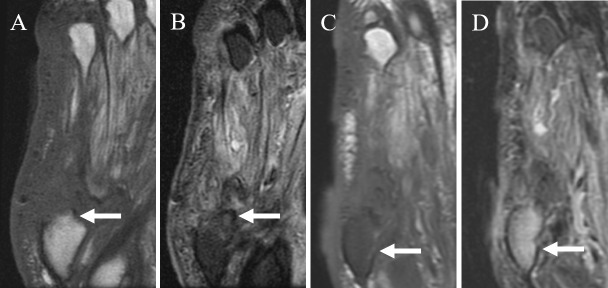
Coronal T1 **(a)** and STIR **(b)** images in a 62-year-old male patient with prior fifth ray amputation and a recurrent ulcer demonstrate a small amount of reticulated T1 hypointense and T2 hyperintense signal near the amputation margin of the fifth metatarsal base (arrows). Follow-up coronal T1 **(c)** and T2 fat-saturated images **(d)** obtained approximately 6 months later demonstrate marked confluent T1 hypointense and T2 hyperintense signal in the residual fifth metatarsal base (arrows) consistent with osteomyelitis.

Among all patients, an ulcer was present in 93 of 112 cases (83.0 %). Among all patients, 89 of 112 (79.5 %) had diabetes; 82 of 89 patients (92.1 %) had type-2 diabetes and 7 of 89 patients (7.9 %) had type 1. Rates of osteomyelitis were not significantly different between those with and without diabetes at either 2 months (37 of 89 patients (41.6 %) vs. 9 of 23 patients (39.1 %); p=1.000 ) or 14 months (41 of 79 patients (51.9 %) vs. 9 of 17 patients (52.9 %); p=1.000 ) post-MRI. There also was no significant difference in the rate of amputation between those with and without diabetes at both the 2-month (32 of 89 patients (36.0 %) vs. 8 of 23 patients (34.8 %); p=1.000 ) and 14-month (45 of 84 patients (53.6 %) vs. 8 of 18 patients (44.4 %); p=0.605 ) time points.

Half (56 of 112) of the patients had a diagnosis of peripheral vascular disease (PVD). Rates of osteomyelitis were not significantly different between those with and without PVD at either 2 months (27 of 56 patients (48.2 %) vs. 19 of 56 patients (33.9 %); p=0.179 ) or 14 months (31 of 56 patients (59.6 %) vs. 19 of 56 patients (43.2 %); p=0.151 ) post-MRI. There was also no significant difference in the rate of amputation between those with and without PVD at both the 2-month (23 of 56 patients (41.1 %) vs. 17 of 56 patients (30.4 %); p=0.324 ) and 14-month (33 of 56 patients (61.1 %) vs. 20 of 56 patients (41.7 %); p=0.074 ) time points.

There was a significant difference in the rate of progression to osteomyelitis at 2 months post-MRI when patients were segregated by antibiotic treatment status ( p=0.02 0): no antibiotics, 3 of 19 patients (15.8 %); oral antibiotics, 7 of 20 patients (35.0 %); and IV antibiotics, 36 of 72 patients (50.0 %). A significant difference was also seen at 14 months post-MRI ( p=0.037 ): no antibiotics, 4 of 15 patients (26.7 %); oral antibiotics, 8 of 18 patients (44.4 %); and IV antibiotics, 38 of 62 patients (61.2 %). No significant difference was seen between these groups with respect to the rate of amputation at either 2 months ( p=0.068 ) or 14 months ( p=0.485 ).

## 4 Discussion

This study examined the association of T1 bone marrow signal on pedal MRI with the subsequent diagnosis of osteomyelitis and amputation. As expected, patients who had confluent T1 hypointense bone marrow signal were most likely to be diagnosed with osteomyelitis and/or require amputation. In these patients, amputation, if it occurred, was almost always required early in the disease course. In patients without confluent T1 hypointense bone marrow signal, however, the majority did not go on to develop osteomyelitis nor require amputation. When amputations were performed in this group, nearly half of them did not occur until after the 2-month follow-up. Although not assessed with the current study, this could be due to factors such as a slower progression of disease and/or interim attempts at conservative therapy.

In the 2017 study by Duryea et al. (2017), 61 % of patients with edema-like signal and reticulated T1 hypointense signal abnormality went on to develop osteomyelitis at 14 months; this value is higher than that in our study in which 38.5 % of patients with reticulated T1 hypointense signal had osteomyelitis at the same time point. One difference is that our study did not require a soft tissue ulcer for inclusion, although it was present in 83 % of cases. In another study of patients with diabetes and suspected osteomyelitis, confluent T1 marrow pattern was the most accurate primary finding of osteomyelitis, whereas reticulated T1 hypointense bone marrow signal was seen in over 90 % of patients without osteomyelitis (Jang et al., 2020).

When MRI is obtained during the evaluation of suspected pedal osteomyelitis, the terminology that is used in the radiology report has the potential to affect clinical management. Based on the findings of this study, the authors suggest avoiding the terms “early osteomyelitis” or “infectious osteitis” for pedal MRI examinations without confluent T1 hypointense bone marrow signal. Although there is clearly a risk of progression (Fig. 5), a large portion of these patients will heal with conservative therapies. Our recommendation is to utilize terminology such as “osteitis,” “reactive osteitis”, or “nonspecific reactive change” when there is bone marrow edema-like signal and reticulated hazy T1 hypointense signal but no associated confluent T1 hypointensity. This approach is similar to that described in an educational article on the use of MRI in pedal osteomyelitis (Donovan and Schweitzer, 2010). This MRI finding of reactive osteitis alone should not lead to altered clinical management, such as biopsy or the initiation of antibiotic therapy.

One limitation to this study is the inherent difficulty in making the diagnosis of osteomyelitis; however, the criteria utilized were similar to prior studies (Duryea et al., 2017; Jang et al., 2020). Histopathologic information is not always available, as it is difficult to culture organisms from bone, and histopathologic diagnosis has poor inter-rater reliability (Meyr et al., 2011). Patients who undergo amputation are often already on aggressive antibiotic therapy, which makes obtaining a positive culture even more challenging. This lack of confirmatory data in some cases may have led to false negative cases based the definition of osteomyelitis utilized for this study. As a result, there are greater numbers of amputations than confirmed diagnoses of osteomyelitis. It is also possible that critical limb ischemia with nonhealing ulcers led to amputation in some cases, rather than osteomyelitis. The inclusion of patients with suspected osteomyelitis at a quaternary medical center introduced selection bias, potentially leading to higher rates of osteomyelitis and amputation in our population. Although MRI is readily available at our medical center, some institutions may rely more heavily on radiographic findings if MRI is not available or financially accessible. In a study of diabetic foot osteomyelitis at one such institution, approximately 5 % of patients had findings of osteomyelitis seen on MRI without radiographic changes, but this was not associated with a significant difference in therapy or remission rate (Gariani et al., 2021). Conventional radiograph is typically the initial imaging test performed at our institution; however, radiographic findings were outside of the scope of the current study and were not included. A total of 10 patients (8.9 %) were lost to follow-up at the 14-month time frame, all occurring in the normal and reticulated T1 signal groups. Finally, the study is limited by its retrospective nature.

## 5 Conclusions

Confluent T1 hypointense intramedullary signal abnormality on MRI is well established as the imaging reference standard for the diagnosis of osteomyelitis. In this study, patients with geographic confluent T1 hypointense intramedullary signal abnormality on pedal MRI were significantly more likely to have confirmed osteomyelitis and require amputation than the other groups. Patients with normal or hazy reticulated T1 hypointense marrow signal abnormality responded to conservative measures over half of the time. Therefore, we recommend the term “osteitis” or “nonspecific reactive change” to describe this hazy reticulated T1 hypointense bone marrow signal abnormality in order to avoid overdiagnosis of osteomyelitis.

## Data Availability

The datasets used and/or analyzed during the current study are available from the corresponding author on reasonable request and after seeking permission from Mayo Clinic.
